# Addiction to Ultra -Processed Foods In the general population

**DOI:** 10.1192/j.eurpsy.2025.956

**Published:** 2025-08-26

**Authors:** N. Kissa, N. Ait Bensaid, A. Korchi, F. Laboudi, A. Ouanass

**Affiliations:** 1 University psychiatric hospital Arrazi; 2 University Psychiatric Hospital Arrazi of Salé Faculty of Medicine and Pharmacy – Mohammed V University of Rabat, Salé, Morocco

## Abstract

**Introduction:**

IIn recent decades, the global rise in obesity and related diseases has led researchers to investigate factors like ultra-processed foods (UPF), known for promoting overconsumption due to their low cost and hyper-palatable design. Studies link UPF to obesity, cardiovascular diseases, and type 2 diabetes, while the concept of food addiction to UPF has emerged to explain compulsive eating. Although not yet officially recognized, UPF addiction calls for urgent public health strategies to regulate their production and consumption.

**Objectives:**

We aim to assess addiction to ultra-processed foods in the general population, its effects on mental and physical health, and explore the factors influencing these eating behaviors to propose intervention approaches for preventing and treating this form of addiction.

**Methods:**

A questionnaire assessed ultra-processed food addiction in the general population, along with its health effects and factors influencing this behavior. A multivariate analysis, including a logistic regression model, measured the impact of different variables on this addiction.

**Results:**

Using the YFAS 2.0 scale, 16.2% of participants show dependence on ultra-processed foods, mostly in mild to moderate forms. Logistic regression reveals that addiction is associated with being female, overweight or obese, and having low perceived well-being, increasing the risk by 6.8, 4.9, and 7.6 times, respectively. These findings suggest that food addiction is influenced by both biological (e.g., BMI) and psychological (e.g., well-being) factors.

**Conclusions:**

The literature shows that addiction to ultra-processed foods, though not officially recognized, is associated with overconsumption behaviors and various health issues, including obesity and cardiovascular diseases. These foods, designed to be highly palatable, activate the brain’s reward circuits. Public health strategies, such as regulation and labeling, are essential to prevent negative health impacts.

**Disclosure of Interest:**

None Declared

